# Serum Presepsin Levels Are Not Elevated in Patients with Controlled Hypertension

**DOI:** 10.1155/2018/8954718

**Published:** 2018-02-08

**Authors:** Ismail Biyik, Fatma Nihan Turhan Caglar, Nilgun Isiksacan, Nursel Kocamaz, Pınar Kasapoglu, Asuman Gedikbasi, Faruk Akturk

**Affiliations:** ^1^Department of Cardiology, School of Medicine, Education and Research Hospital, Uşak University, Uşak, Turkey; ^2^Department of Cardiology, Bakirkoy Dr. Sadi Konuk Education and Research Hospital, Istanbul, Turkey; ^3^Department of Biochemistry, Bakirkoy Dr. Sadi Konuk Education and Research Hospital, Istanbul, Turkey; ^4^Department of Internal Medicine, Bakirkoy Dr. Sadi Konuk Education and Research Hospital, Istanbul, Turkey

## Abstract

**Introduction:**

Hypertension (HT) is a common serious condition associated with cardiovascular morbidity and mortality. The pathogenesis of HT is multifactorial and has been widely investigated. Besides the vascular, hormonal, and neurological factors, inflammation plays a crucial role in HT. Many inflammatory markers such as C-reactive protein, cytokines, and adhesion molecules have been studied in HT, which supported the role of inflammation in the pathogenesis of HT. Presepsin (PSP) is a novel biomarker of inflammation. Therefore, the potential relationship between PSP and HT was investigated in this study.

**Methods:**

Forty-eight patients with controlled HT and 48 controls without HT were included in our study. Besides routine clinical and laboratory data, PSP levels were measured in peripheral venous blood samples from all the participants.

**Results:**

PSP levels were significantly lower in patients with HT than in controls (144.98 ± 75.98 versus 176.67 ± 48.12 pg/mL, *p* = 0.011). PSP levels were positively correlated with hsCRP among both the patient and the control groups (*p* = 0.015 and *p* = 0.009, resp.). However, PSP levels were not correlated with WBC among both groups (*p* = 0.09 and *p* = 0.67, resp.).

**Conclusions:**

PSP levels are not elevated in patients with well-controlled HT compared to controls. This result may be associated with anti-inflammatory effects of antihypertensive medicines.

## 1. Introduction

The number of people living with hypertension (HT) worldwide has been estimated to be 1.56 billion by the year 2025 [1]. HT is basically defined as increased peripheral vascular resistance to blood flow [2]. The underlying pathophysiology has been widely investigated and is now considered as multifactorial and complex [3]. Besides other vascular, humoral, or endothelial factors, inflammation plays a role in the development and progression of HT [3]. The association between C-reactive protein (CRP), tumor necrosis factor-alpha (TNF-*α*), interleukin-6 (IL-6), and other adhesion molecules and HT is an indicator of the role of inflammation during HT setting [4]. Although there are an enormous number of studies conducted on HT, it is still a major health problem worldwide. Novel inflammatory markers have been investigated in order to help guide future therapeutic targets. Presepsin (PSP) is a novel inflammatory marker recommended as an acute phase reactant similar to CRP [5, 6]. PSP is a glycoprotein that is split out from the monocyte/macrophage-specific cluster of differentiation (CD) subtype 14 N-terminal [5, 6]. CD-14 is one of the receptors of lipopolysaccharide (LPS)/LPS-binding protein (LBP) complexes [5, 7]. PSP is truncated from this receptor complex during inflammation [6]. The diagnostic and predictive importance of circulating PSP levels is mostly investigated during severe inflammatory situations [5, 7]. The aim of this study was to investigate the relationships between PSP levels and well-controlled HT in patients with primary HT.

## 2. Materials and Methods

### 2.1. Study Population

This observational comparative study was conducted in a tertiary referral center. We followed the methods of Caglar et al. (2017) [8]. Forty-eight well-controlled hypertensive patients with primary HT (patient group) and a healthy voluntary control group of 48 patients without HT (control group) were enrolled in the study. The study protocol was approved by the local ethics committee review board. The study complied with the Declaration of Helsinki and voluntary informed written consent was obtained from all patients included in this study. The patient group consisted of patients with grade 1-2 primary HT. HT was defined and graded according to the European Society of Cardiology and European Society of Hypertension guideline [9]. Patients with secondary HT, grade 3 HT, and malign HT were excluded from the study. The control group consisted of healthy volunteers. All clinical available data at the time of initial visit were collected by two cardiologists from the medical records of each patient. A previous diagnosis of diabetes mellitus (DM), the use of antidiabetic medicines, and a fasting venous blood glucose level of 126 mg/dL on two occasions in previously untreated patients were required for the diagnosis of DM. The glomerular filtration rate was estimated using the MDRD (Modification of Diet in Renal Disease) equation at admission. Patients with known inflammatory disease, estimated glomerular filtration rate (eGFR) < 60 mL/min/1.73 m^2^, serious valvular heart disease, heart failure, serious hepatic failure, acute or chronic infection, fever, muscle aches, headaches, immunoproliferative disease, rheumatic disease, malignancy, and osteoporosis; those under 18 years of age and above 70 years of age; and those receiving antibiotics therapy were also excluded from the study [8].

### 2.2. Laboratory Measurements

All of the patients' laboratory data such as creatinine, white blood cell (WBC) count, and high sensitive CRP (hsCRP) were documented. Blood samples for PSP were drawn just after randomization. Blood samples were obtained by vein puncture into ethylenediaminetetraacetic acid (EDTA) blood collection tubes without additives and immediately centrifuged at 2500 rpm for 10 minutes [8]. The serum was collected after centrifugation and stored at −80°C until analysis up to 6 months and the samples were thawed out once [8]. All the assays were performed on serum according to the manufacturer's recommendations with the PATHFAST® immunoassay analytical system (Progen Biotechnik GmbH, Germany; Mitsubishi Chemical Medience Corporation, Japan) using plasma from EDTA tubes [8].

### 2.3. Statistical Analysis

Number Cruncher Statistical System (NCSS) (Kaysville, Utah, USA, 2007) program was used for statistical analysis. Study data were analyzed using descriptive statistical methods such as mean, standard deviation, median, frequency, ratio, minimum, and maximum. In the analysis, Student's *t*-test was used for normally distributed quantitative data, and Mann–Whitney *U* test was used for non-normally distributed data [8]. Comparisons of qualitative data were analyzed by Fisher's Exact Test. Spearman's rank correlation was used to test the correlations among data. Two-tailed *p* values lower than 0.01 with 99% confidence level and 0.05 with 95% confidence level were considered statistically significant.

## 3. Results

**Figure 1:**
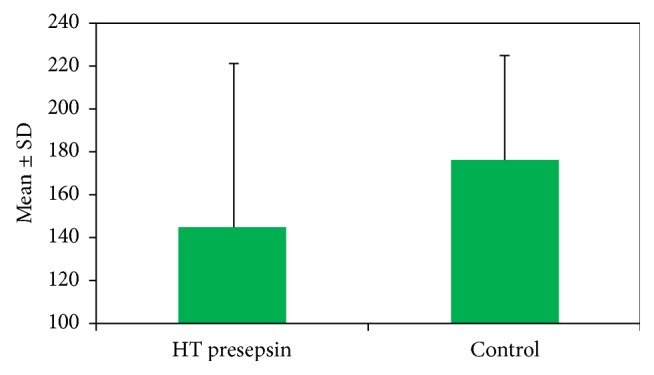
Presepsin levels among groups.

Forty-eight patients with HT (11 (22.9%) males, 37 (77.1%) females) and 48 controls without HT (20 (31.5%) males, 28 (58.3%) females) were enrolled in the study. Baseline characteristics and laboratory findings are given in Table 1. Accordingly, smoking and medical history were similar among groups. PSP levels were significantly lower in the HT group than in controls (144.98 ± 75.98 versus 176.67 ± 48.12 pg/mL, *p* = 0.011) (Figure 1). hsCRP levels were similar among groups (0.87 ± 1.61 versus 0.9 ± 0.55 mg/L, *p* = 0.137). Creatinine levels were similar among groups (0.78 ± 0.17 versus 0.87 ± 0.25 mg/dl, *p* = 0.056). White blood cell (WBC) count was similar among groups (8.44 ± 2.41 versus 8.89 ± 2.27 × 10^9^/L, *p* = 0.58). Correlation analyses of biomarkers among groups are given in Table 2. Accordingly, PSP levels were positively correlated with hsCRP among both the patient and the control groups (*p* = 0.015 and *p* = 0.009, resp.). However, PSP levels were not correlated with WBC among both groups (*p* = 0.09 and *p* = 0.67, resp.). The distribution of antihypertensive medications in the patient group is given in Table 3. Accordingly, most of the HT patients were taking angiotensin-converting enzyme inhibitors or angiotensin receptor blockers (ACE-i/ARB), and others were taking beta-blockers (BB), calcium channel blockers, and diuretics.

## 4. Discussion

PSP levels were not elevated in patients with primary HT compared to healthy controls. Although PSP values in both groups were within normal limits, PSP levels were statistically significantly lower in patients with controlled HT than in the control group. Our results may seem negative at first. However, our work is actually coherent with previous knowledge saying that HT is a condition of chronic low-grade inflammatory status rather than a highly fatal acute state [4]. On the other hand, PSP is a sensitive and specific marker for high-grade inflammation [10]. Normal healthy blood naturally has a small amount of PSP for activation of endothelial and epithelial cells by LPS and its serum levels increase in response to inflammation [11, 12]. Previous studies reported normal serum PSP levels within a wide range from 55 to 600 pg/mL [11–13]. Subject selection bias and/or the PSP measurement method may be the reason of this wide range [11–13]. We used the chemiluminescent enzyme immunoassay method for PSP measurement, which is the most accepted method in related studies. PSP levels were 144.98 ± 75.98 pg/mL in the HT group and 176.67 ± 48.12 pg/mL in the control group. Thus, it may be suggested that PSP levels were in the normal range in both groups. Recent studies have evaluated the sensitivity and specificity of PSP in various clinical conditions [8, 10, 14–19]. Hou et al. stated that PSP is a sensitive predictor of inflammation in patients with nephrolithiasis and that it can also be used as a monitoring marker [14]. Endo et al. evaluated the predictive value of PSP during sepsis and found PSP to be more valuable than blood culture [15]. Popov et al. studied the prognostic value of PSP in patients operated on for acquired heart diseases and revealed that PSP levels were increased in patients operated on with acute HF and acute coronary syndrome without infection [16]. Shozushima et al. demonstrated that PSP had higher clinical specificity than procalcitonin for the diagnosis of infections [17]. Presepsin levels may be correlated with the severity of the illness. Klouche et al. investigated PSP in patients with severe sepsis, septic shock, and severe community-acquired pneumonia and demonstrated a different amount of PSP increase among subgroups, which was correlated with the severity of the illness [18]. Masson et al. stated using PSP as an early risk stratification tool in patients with severe sepsis after showing significantly higher PSP levels in patients who died of severe sepsis than in patients who survived [19]. Olad et al. demonstrated that increased PSP levels in patients with chemotherapy induced severe neutropenia and although PSP was not sensitive enough to detect culture negative bacteremia, it was significantly higher in patients with culture positive infections [10]. Recently, PSP levels were found to be significantly elevated in patients with acute ST elevation myocardial infarction together with high-sensitivity troponins and Presepsin may be a novel supporting predictor for acute myocardial infarction detection [8]. PSP is a small 13 kDa protein metabolized by the kidneys [20]. PSP is filtered by the glomerulus, reabsorbed, and catabolized by proximal tubular cells [20]. Therefore, PSP levels are elevated during kidney failure [20]. Behnes et al. demonstrated the positive correlation between PSP and creatinine levels [6]. Nagata et al. studied the relationship between normal circulating PSP levels and different stages of chronic kidney disease and demonstrated the negative correlation between PSP and eGFR [11]. Therefore, we did not include patients with eGFR lower than 60 mL/min/1.73 m^2^. PSP levels may also be affected by advanced age. Chenevier-Gobeaux et al. showed significantly increased PSP levels in patients above 70 years of age compared to patients below 70 years old [20]. Therefore, we did not include patients above 70 years of age. Recently, Bomberg et al. reported that elevated preoperative plasma presepsin concentration is an independent predictor of postoperative mortality in elective cardiac surgery patients and they have also emphasized that PSP is a stronger predictor than several other commonly used assessments such as cystatin C, N-terminal prohormone brain natriuretic peptide, and procalcitonin [21]. All of the patients in the HT group were receiving antihypertensive treatment in our study. 66.6% of the patients were taking ACE-i/ARB medication, 29.1% were taking BB, and 20.8% were taking CCB in the HT group. Independent of their blood pressure lowering effect, most of the antihypertensive medicines, especially ACE-i, ARB, CCB, and BB, have been shown to reduce vascular inflammation [22–24]. Although PSP levels were within the normal range in both groups, they were statistically significantly lower in the controlled HT group than in the control group (*p* = 0.011). The present study does have some important limitations. It was a small, single-centered, observational study. We only included patients with grade 1 and 2 HT and all of the patients were receiving antihypertensive treatment. In our opinion, our results may partly be explained with the anti-inflammatory effects of the antihypertensive medicines used in the treatment of the disease. The findings and the hypothesis should be examined intensively, and the study should be extended by including a higher number of patients and by adding other suitable inflammation markers. To our knowledge, this is the first study evaluating PSP levels in patients with HT. Our results are substantially compatible with previous reports suggesting PSP as an acute serious inflammatory marker, whereas HT is a chronic low intensity inflammatory state [4, 5]. Further study recruiting a larger number of hypertensive patients naive to treatment will be needed.

## 5. Conclusion

The present study suggests that PSP levels are not elevated in patients with HT under antihypertensive treatment. This result may be associated with the anti-inflammatory effects of the antihypertensive medicines. Large-scale studies are needed to reveal strong comments.

## Figures and Tables

**Table 1:** Demographic features and laboratory findings of HT and control group.

	HT group (*n* = 48)	Control group (*n* = 48)	*p* value
Age (years), mean ± SD	58.29 ± 11.27	48.94 ± 15.26	0.021
Sex, *n* (%)			
Male	11 (22.9)	20 (31.5)	0.22
Female	37 (77.1)	28 (58.3)	
Smoking, *n* (%)	24 (48.0)	32 (66.7)	0.615
Diabetes, *n* (%)	11 (22.0)	10 (19.6)	0.959
History of CVA, *n* (%)	1 (2.0)	0 (0)	0.495
Presepsin (pg/mL), mean ± SD	144.98 ± 75.98	176.67 ± 48.12	0.011
hsCRP (mg/L), mean ± SD	0.87 ± 1.61	0.9 ± 0.55	0.137
Creatinine (mg/dl), mean ± SD	0.78 ± 0.17	0.87 ± 0.25	0.056
WBC (×10^9^/L), mean ± SD	8.44 ± 2.41	8.89 ± 2.27	0.424

HT: hypertension; SD: standard deviation; hsCRP: high sensitive C-reactive protein; WBC: white blood cells.

**Table 2:** Correlation analysis between presepsin and other inflammatory markers.

	HT group	Control group	Total
	Presepsin	Presepsin	Presepsin
hsCRP			
*r*	0.350	0.594	0.452
*p*	0.015^*∗*^	0.009^*∗∗*^	0.001^*∗∗*^
WBC			
*r*	0.241	0.105	0.255
*p*	0.099	0.677	0.038^*∗∗*^

HT: hypertension; WBC: white blood cells; hsCRP: high sensitive C-reactive protein; *r*: Spearman's correlation coefficient; ^*∗*^*p* < 0.05, ^*∗∗*^*p* < 0.01.

**Table 3:** Distribution of patients in antihypertensive medicine groups.

Drug groups	*n* (%)
ACE-i/ARB	32 (66.6)
BB	14 (29.1)
CCB	10 (20.8)
Diuretics	18 (37.5)

ACE-i: angiotensin-converting enzyme inhibitors; ARB: angiotensin receptor blockers; BB: beta-blockers; CCB: calcium channel blockers.
